# Validation of Roche immunoassay for severe acute respiratory coronavirus 2 in South Africa

**DOI:** 10.4102/sajid.v36i1.286

**Published:** 2021-07-26

**Authors:** Jurette S. Grove, Elizabeth S. Mayne, Wendy A. Burgers, Jonathan Blackburn, Sarika Jugwanth, Wendy Stevens, Lesley Scott, Anura David, Maemu Gededzha, Ian M. Sanne, Mpho R. Maphayi, Taryn Pillay, Jaya A. George

**Affiliations:** 1 Department of Chemical Pathology, Faculty of Health Sciences, University of the Witwatersrand, Johannesburg, South Africa; 2 National Health Laboratory Service, Johannesburg, South Africa; 3 Department of Immunology, Faculty of Health Sciences, University of the Witwatersrand, Johannesburg, South Africa; 4 Institute of Infectious Diseases and Molecular Medicine, Faculty of Health Sciences, University of Cape Town, Cape Town, South Africa; 5 Department of Virology and Wellcome Centre for Infectious Diseases Research in Africa, University of Cape Town, Cape Town, South Africa; 6 Division of Chemical and Systems Biology, Department of Integrative Biomedical Sciences, University of Cape Town, Cape Town, South Africa; 7 Department of Molecular Medicine and Haematology, Faculty of Health Sciences, University of the Witwatersrand, Johannesburg, South Africa; 8 HIV Research Unit, Faculty of Health Sciences, University of the Witwatersrand, Johannesburg, South Africa

**Keywords:** COVID-19, SARS-CoV-2, serology, antibodies, validation, immunoglobulin G, immunoglobulin M

## Abstract

**Background:**

Serology testing is an important ancillary diagnostic to the reverse transcriptase polymerase chain reaction (RT-PCR) test for severe acute respiratory syndrome coronavirus 2 (SARS-CoV-2). We aimed to evaluate the performance of the Roche Elecsys™ chemiluminescent immunoassay (Rotkreuz, Switzerland), that detects antibodies against the SARS-CoV-2 nucleocapsid antigen, at an academic laboratory in South Africa.

**Methods:**

Serum samples were collected from 312 donors with confirmed positive SARS-CoV-2 RT-PCR tests, with approval from a large university’s human research ethics committee. Negative controls included samples stored prior to December 2019 and from patients who tested negative for SARS-CoV-2 on RT-PCR and were confirmed negative using multiple serology methods (*n* = 124). Samples were stored at –80 °C and analysed on a Roche cobas™ 602 autoanalyser.

**Results:**

Compared with RT-PCR, our evaluation revealed a specificity of 100% and overall sensitivity of 65.1%. The sensitivity in individuals > 14 days’ post-diagnosis was 72.6%, with the highest sensitivity 31–50 days’ post-diagnosis at 88.6%. Results were also compared with in-house serology tests that showed high agreement in majority of categories.

**Conclusions:**

The sensitivity at all-time points post-diagnosis was lower than reported in other studies, but sensitivity in appropriate cohorts approached 90% with a high specificity. The lower sensitivity at earlier time points or in individuals without symptomatology may indicate failure to produce antibodies, which was further supported by the comparison against in-house serology tests.

## Introduction

The coronavirus disease 2019 (COVID-19) is a viral infection caused by severe acute respiratory coronavirus 2 (SARS-CoV-2), a novel coronavirus first identified in Wuhan, China in December 2019. It has subsequently caused a global pandemic, infecting more than 126 million people and resulting in the death of more than 2.7 million individuals worldwide.^1,2^

Whilst non-pharmacological methods such as social distancing can limit the spread of the disease, there is a need for rapid identification of infected individuals not only for diagnosis but also to prevent further transmission.^3^

The gold standard for acute SARS-CoV-2 diagnosis is the reverse transcriptase polymerase chain reaction (RT-PCR) performed on an oropharyngeal or nasopharyngeal swab sample. The acceptable turnaround time for this test in South Africa is 24 h – 48 h, but the large burden of disease and worldwide shortage of test kits have constrained availability, resulting in prolonged turnaround times worldwide.^4^ Further limitations of RT-PCR include that detection relies on the presence of the viral genome in sufficient amounts for amplification.^5^

Missing the window of viral replication and incorrect sampling may produce false-negative results.^5,6^ There is therefore a demand for additional testing strategies.

Serological tests that identify antibodies produced in response to infection have the potential for a rapid turnaround time.^7^ Although the extent and timing of the humoral response against SARS-CoV-2 is still under investigation, immunoglobulin M (IgM) and A (IgA) antibodies directed at one or more of the major structural proteins (membrane, envelope, spike and nucleocapsid) are generally detectable at a median of day 5 and immunoglobulin G (IgG) antibodies at a median of day 14 post-symptom onset.^5^ Immunoglobulin M antibody levels drop after about day 14 when the IgG antibody levels start to rise. There is potential for serological assays to be utilised in a variety of ways: to assist with diagnosis of the disease together with RT-PCR,^8^ to identify past infections including in paediatric patients with multisystem inflammatory syndrome of COVID (MIS-C),^9^ to perform seroprevalence studies,^4^ to assess the immune response to a potential COVID-19 vaccine and, lastly, to identify donors for convalescent plasma.^10^

Since the identification of serology methods as an ancillary diagnostic, there has been a rapid development of a wide range of different assays.^4^

A review of 40 studies indicated that chemiluminescent immunoassays (CLIA) methodology had the highest sensitivity with a pooled sensitivity of 97.8%, compared with enzyme linked immunosorbent assay (ELISA) with 84.3% and lastly lateral flow immunoassays (LFIA) with 66.0%.^11^ Roche Diagnostics (Rotkreuz, Switzerland) developed an electrochemiluminescent immunoassay (Elecsys™ Anti-SARS-CoV-2), which detects total antibodies against the SARS-CoV-2 nucleocapsid antigen, although it is most specific for IgG and IgM. Results are reported in a qualitative manner: below the cut-off index (COI) of 1 is interpreted as non-reactive, compared with a reactive result that is equal or greater than the COI of 1.

The manufacturer claims that this assay has a specificity of 99.81%, with a 100% sensitivity after day 14.^12^ There have been multiple validations of the assay as set out in Table 1. Importantly, sensitivity is generally reported at time points postinfection of 14 days or more when IgG antibodies are more likely to be produced.

**TABLE 1:** Cumulative review of validations of Roche Elecsys™ severe acute respiratory virus syndrome coronavirus 2 assay.

Location	Number of positive samples included	Total number of positive participants	Number of negative controls	Reported sensitivity > 14 days post-positive RT-PCR (%)	Reported specificity (%)	Reference
Belgium	140	97	79	91.1	100.0	Favresse et al.^13^
Singapore	349	205	715	97.1	99.9	Lau et al.^14^
Germany	186	58	88	89.1	100.0	Hörber et al.^15^
Taiwan	346	74	194	97.4 (> 21 days)	99.0	Chen et al.^16^

RT-PCR, reverse transcriptase polymerase chain reaction.

Limitations of many of these evaluations are that numbers of positive participants sampled were small and individuals tested were predominantly symptomatic. Their performance has also not been extensively examined at time points postinfection of 30 days or more raising questions regarding the persistence of an immunological response to the virus.

Of note, there have been limited validations in the African context. International studies suggest that patients of African descent are disproportionately likely to have severe disease and to die.^17^ South Africa is currently the epicentre of the African pandemic with 1 545 979 cases and 52 710 deaths (29 March).^18^ This prompted the evaluation of ancillary diagnostic methods in the African setting.

The auto-laboratory at Charlotte Maxeke Johannesburg Academic Hospital (CMJAH) and the National Health Laboratory Service (NHLS) is a large tertiary referral laboratory servicing the Johannesburg area and surroundings. This laboratory operates a Roche cobas 602 (Rotkreuz, Switzerland). Our aim was to validate the Roche Elecsys™ electrochemiluminescent immunoassay and to assess the immune response based on symptomatology and number of days’ post-molecular diagnosis and to identify the appropriate use case of this testing in South Africa.

## Material and methods

### Subjects

This prospective analytical study was conducted at the NHLS based at a large tertiary hospital’s laboratory between May and August 2020. Patients who tested positive for SARS-CoV-2 with RT-PCR on a nasopharyngeal swab within South Africa were invited to take part in the study. At the time of this study, there was a global shortage of RT-PCR reagents because of the increased demand for testing. As a result of the aforementioned problem, it was not possible to include RT-PCR results utilising only one uniform reagent. Instead, positive RT-PCR that utilised one of the following reagents were included: Allplex™ 2019 nCoV assay (Seegene, Korea) that targets E, ribonucleic acid-dependant polymerase (RdRP) and N genes; TaqPath™ COVID-19 V2 assay (Applied Biosystems by ThermoFisher Scientific, United States of America) that targets open reading frame of 1ab (ORF1ab), S and N genes; LightMix® Modular SARS and Wuhan CoV E-gene kit (TIB Molbiol for Roche Diagnostics, Switzerland) that targets RdRp and E genes and lastly the Abbott Alinity m SARS-CoV-2 assay (Abbott Molecular, United States) that targets N and RdRP gene. A positive RT-PCR was defined as two or more gene targets identified as positive, with a cycle threshold (Ct) of ≤ 37, as these samples were collected before the National Institute of Communicable Diseases’ (NICD) guideline to report a positive result if one or more gene targets were identified as positive. Majority of the positive RT-PCR samples, and specifically all samples where uncertainty existed about the method that was used, had a repeat confirmatory test conducted on the same sample on the Gene Xpert (Cepheid, Sunnyvale, United States) platform within 7 days. Samples were stored for 7 days at –80 °C, and samples that could not be verified were excluded from the study. Venous blood samples were obtained after consent in a serum separator or Ethylenediaminetetraacetic acid (EDTA) tube (BD Vacutainer™) because both serum and plasma were acceptable for the platform. After centrifugation, plasma or serum was extracted, aliquoted and subsequently frozen at –80 °C, with freeze-thaw cycles limited to one. Negative control samples included remnant samples from patients stored prior to December 2019 and from patients who tested negative for SARS-CoV-2 on RT-PCR, confirmed to be negative on other serology methods, particularly in-house anti SARS-CoV-2 ELISAs, to mitigate the risk of using a false negative sample.

Data were also collected on the age and symptomatology of participants, if available and consented.

### Methods

For this study we investigated the Roche Diagnostics Elecsys™ Anti-SARS-CoV-2 electrochemiluminescent immunoassay (Rotkreuz, Switzerland) that detects total antibodies against SARS-CoV-2.

Evaluation of the analytical performance was carried out in accordance with the Clinical and Laboratory Standards Institute (CLSI) EP 12 document and the United States’ Food and Drug Administration (FDA) guidelines.^19,20^

Initial assay optimisation was performed on 93 patient samples prior to commencing with the full clinical validation. In the absence of standardised quality control (QC) material commercially available at the time, positive controls were derived from three positive pooled patient samples and negative controls from five negative pooled samples, as recommended by the manufacturer.

**FIGURE 1:**
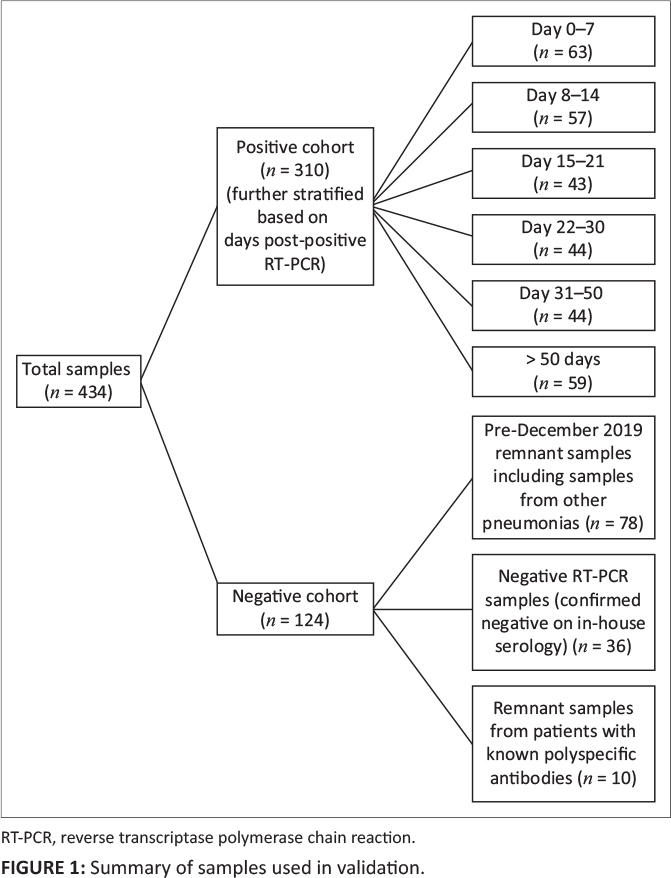
Summary of samples used in validation.

The clinical validation consisted of 434 participants’ samples. This included negative samples (*n* = 124) and positive samples (*n* = 310). The positive samples were further stratified based on the number of days post RT-PCR diagnosis (Figure 1).

Samples were thawed and run on the Roche cobas™ e602 module. Although the risk of a false positive RT-PCR was mitigated by repeating analysis on the Gene Xpert platform, as discussed here, comparison against RT-PCR as the gold standard is inherently flawed because a positive RT-PCR does not guarantee a positive serology result. To address the potential misclassification of samples as false negatives, that were in fact true negatives in patients with positive RT-PCR that did not produce antibodies, a comparison was performed against a composite serology platform consisting of a Western blot and immunofluorescence, that utilised insect cell-expressed recombinant full length N and S antigens and an in-house serology ELISA assay utilising plant-based recombinant spike 1 (S1) and receptor binding domain (RBD) antigens, in collaboration with another large university.^21^ After completion on the Roche platform, all samples were sent for analysis on the composite serology platform. Unfortunately, because of sample volume constraints, only 216 out of the 434 samples had sufficient results on the composite serology platform to form part of the comparison against this method. This consisted of 33 negative samples and 183 positive samples. A result was deemed as positive if 2/3 or 3/3 methods yielded a positive result.

Inter-run precision was carried out in line with the CLSI recommendation, although it had to be modified to run over 3 days instead of 5 because the on-board stability of the reagent at the time of validation was only 72 h.

### Statistical analysis

Results were compared with both the RT-PCR and disaggregated by days post-diagnosis. Samples were further stratified by symptom score, which was assigned as follows: 0 for asymptomatic, 1 for mild diseases indicating only respiratory tract symptoms, 2 for moderate disease indicating symptoms outside of the respiratory tract and 3 for severe disease requiring admission. Samples were further compared with in-house serology, to assess the presence of detectable antibodies of results because the limitations of RT-PCR are well known (including the ability to detect past infection).

### Data analysis

Statistical analysis was performed with MedCalc statistical software version 19.4.1 (MedCalc Software Ltd., Belgium). We defined sensitivity as the proportion of correctly identified COVID-19-positive patients who were positive by RT-PCR SARS-CoV-2 analysis in respiratory samples, whilst specificity was defined as the proportion of correctly identified negative samples. Sensitivity was also reported according to symptom score and days post positive RT-PCR.

Inter-run precision was calculated with Microsoft Excel as CV (%) = (standard deviation [SD] × 100)/mean, and expressed as a percentage coefficient of variation.

### Ethical considerations

Ethical approval to conduct the study was obtained from the Human Research Ethics Council of the University of the Witwatersrand (reference number: M200694). Informed consent was obtained from all participants.

## Results

The median age of both cohorts was 42, the positive cohort (*n* = 310) with a range of 19–87 years (minimum to maximum), compared with the negative control (*n* = 124) of 22–75 years. Both cohorts had a slight female preponderance (61.6% in the positive group and 57.1% in the negative group). By symptom category, moderately symptomatic participants were the largest group (*n* = 90) and asymptomatic participants were the smallest group (*n* = 33). Participants were almost equally distributed by number of days post diagnosis, except for those between 0 and 7 days who made up 20% of the total cohort (*n* = 63).

### Accuracy analysis

The specificity of the assay was high at 100% (95% confidence interval [CI] 97.07% – 100%). The overall sensitivity of the Roche assay across all participants was 65.2% (95% CI 59.57% – 70.46%) when compared with RT-PCR results.

Samples were also stratified by days post RT-PCR and degree of symptoms and compared with both the RT-PCR and the in-house composite serology platform (Table 2). Comparison against RT-PCR demonstrated that sensitivity was greatest at > 14 days post PCR in severely symptomatic participants. Comparison against in-house serology demonstrated much higher agreement in all groups, with 100% > 30 days post positive RT-PCR.

**TABLE 2:** Sensitivity of stratified data – Days post positive reverse transcriptase polymerase chain reaction and symptoms, compared to reverse transcriptase polymerase chain reaction and agreement compared with composite serology.

Stratified data days post positive RT-PCR	Sensitivity compared to RT-PCR (%)	95% confidence interval (%)	Agreement compared with composite serology (%)	95 % confidence interval (%)
0–7 days	52.4	39.4–65.1	84.6	54.6–98.1
8–14 days	54.4	40.7–67.6	77.8	40–97.2
15–21 days	72.1	56.3–84.7	88.2	63.6–98.5
22–30 days	47.7	32.5–63.3	66.7	34.9–90.1
31–50 days	88.6	75.4–96.2	100.0	86.3–100
> 14 days	72.6	65.7–78.8	93.3	85.9–97.5
> 50 days	79.7	75.4–96.2	100.0	86.3–100
**Symptom score**				
0 (asymptomatic)	57.6	39.2–74.5	81.8	48.2–97.7
1 (mildly symptomatic)	59.2	44.2–73	82.6	61.2–95
2 (moderately symptomatic)	64.4	53.7–74.3	91.7	80–97.7
3 (severely symptomatic)	69.8	57–80.7	90.9	58.7–99.8
**Stratified data – Days post-PCR in symptomatic individuals**				
> 14 days in moderately to severely symptomatic	81	70.6–89	97.6	87.4–99.9

RT-PCR, reverse transcriptase polymerase chain reaction.

Compared with the in-house serology, the overall positive agreement was 89.4% (95% CI 82.18% – 94.39%), with a slight reduction in negative agreement of 88.4% (95% CI 80.53% – 93.83%). The asymptomatic group demonstrated the lowest sensitivity or agreement across both comparisons.

As the asymptomatic group revealed the lowest sensitivity, analysis of the data was repeated with removal of asymptomatic patients from all cohorts, which revealed an increase in the sensitivity in all the groups > 14 days, apart from a slight decline in > 50 days, as set out in Table 3.

**TABLE 3:** Sensitivity with the asymptomatic cohort removed.

Stratified data days post positive RT-PCR (days)	Sensitivity compared with RT-PCR without the asymptomatic cohort (%)	95% confidence interval (%)
0–7	47.6	32–63.6
8–14	50	34.2–65.8
15–21	85.7	67.3–95.8
22–30	61.3	42.2–78.2
31–50	90	73.5–97.9
> 14	76.5	67.7–83.9
> 50	70	48.2–85.7

RT-PCR, reverse transcriptase polymerase chain reaction.

### Precision analysis

In order to assess inter-run repeatability, 98 of the samples were tested in duplicate, with a qualitative repeatability result of 100%. Ten samples with results around the COI of one were identified and also tested in duplicate, with 100% agreement.

The QC material, made up as recommended by the manufacturer, was tested five times over 3 days to accommodate the short on-board reagent stability. The assay showed acceptable precision with an index value of 0.093 and a coefficient of variation of 1.5% in the negative controls, and an index value of 3.14 and a coefficient of variation (CV) of 2.2% in the positive controls. This broadly agrees with the % CV of other immunoassays performed on the cobas e602.^14,22^

Samples from patients with known autoimmune disease and poly-specific immunoglobulins, obtained before February 2020, were also analysed (*n* = 10), with 100% specificity. Seven of these samples were tested in duplicate, with 100% result concordance.

## Discussion

This study aimed to evaluate the test performance of the Roche Elecsys Anti-SARS-CoV 2 assay tested on a cobas 602 in a high prevalence setting in South Africa. Results were compared with RT-PCR as the gold standard. This revealed a variable sensitivity ranging from 47.8% to 88.6% and a high specificity of 100%, the latter in line with the manufacturer’s claims.^12^

These findings support the use of this assay for the case proposed including for retrospective diagnosis and for seroprevalence studies because a high diagnostic specificity is essential in these settings. There was no cross-reactivity observed with samples that had poly-specific antibodies. Sensitivity was moderately low in individuals more than 14 days’ post-positive test result at 72.63%, but improved in patients with moderate or severe symptoms. This test demonstrated the highest sensitivity compared with RT-PCR in severely ill or admitted individuals at time points of 14 days post-diagnosis although this failed to reach a level of 100% sensitivity after 14 days in any group as claimed by the manufacturer.^12^ This study found low sensitivity of the assay in asymptomatic patients and in patients before 14 days post-diagnosis, which agrees with findings from other validations internationally. This reflects the dynamics of the humoral response to SARS-CoV-2, which suggests that IgG antibodies are only detectable on day 10 post-infection and reach a maximum after day 14 and IgM antibodies are produced at about day 5–7 but are transient and are not produced by all patients.^5,13,15,23^ This is further supported by the high agreement in these time points compared with composite serology. There was a slight decline in sensitivity seen at day 50, but this was insignificant. Although few studies of antibody persistence have been published, there are suggestions in some cases that antibody levels may decline after 2 months and that IgM particularly is undetectable after 30–60 days in most patients.^24,25^ The poor performance in day 22–30 post diagnosis could not be fully explained, although only a third of the patients with known symptomatology were classified as severe, which may have skewed the data in this participant group. This was supported by the increase in sensitivity seen after reanalysis without the asymptomatic patients in the group, although an important confounder remained that symptomatology was not known for a fifth of the group. Importantly, sensitivity remained low when compared with the in-house ELISA and the possibility exists that this indicates that anti-N antibodies were not produced in this subset.

Previous studies have shown that antibodies against the N- and S-protein are produced more or less at the same time, however there is a need for further data to evaluate if antibodies against both are produced in all subjects.^23^

This study is the most extensive evaluation of the Roche Elecsys Anti SARS-CoV-2 electrochemiluminescence immunoassay, assessing all time points post-positive RT-PCR, but most specifically more than 21 days post-positive diagnosis and correlating results with symptomatology. The assay had a high specificity in line with global validations and manufacturer claims. This validation showed the lowest sensitivity in the asymptomatic symptom group, which seems to confirm that not all individuals infected with SARS-CoV-2 produce systemic antibodies, especially if asymptomatic.^26^ Cumulative sensitivity and sensitivities at all the time points compared with RT-PCR as gold standard, were lower than other comparable studies reported globally, but the inclusion of asymptomatic participants in all groups may offer a partial explanation for this decline.^13,14,15,16^ The agreement in all groups significantly improved with comparison against in-house serology. Importantly, the viral and humoral response dynamics indicate that these two assays are sensitive at different time points in infection with the RT-PCR more sensitive prior to day 14 and the serology more sensitive thereafter. This supports the use of these tests for the indications that are approved in South Africa including for retrospective diagnosis of cases where the RT-PCR test was either not performed at the correct time or was falsely negative. This is particularly important in individuals with delayed complications of SARS-CoV-2 infection including so-called long COVID-19 and MIS-C.^9^ Although an incorrect RT-PCR result remains a possibility, this was mitigated by repeating results in house for the majority of the participants.

Limitations of this study included that symptomatology was not described in all of the positive participants. At the time of validation, the short reagent stability and lack of standardised QC material were also limitations. This has, however, been optimised by the manufacturer in more recent lots. This study does provide reassurance that, in a subset of patients, there is acceptable performance that would justify use of this test. We would recommend it is best utilised for retrospective diagnosis in individuals more than 14 days’ post-positive PCR who are moderately or severely symptomatic, as an ancillary diagnostic in multisystem inflammatory disorders and seroprevalence studies.^4,27^
